# Barriers to college student food access: a scoping review examining policies, systems, and the environment

**DOI:** 10.1017/jns.2024.25

**Published:** 2024-09-26

**Authors:** Matthew J. Landry, Rebecca L. Hagedorn-Hatfield, Victoria A. Zigmont

**Affiliations:** 1 Department of Population Health and Disease Prevention, Joe C. Wen School of Population & Public Health, University of California, Irvine, Irvine, CA, USA; 2 Department of Nutrition, Health and Human Performance, Meredith College, Raleigh, NC, USA; 3 Center for Nutrition and Health Impact, Omaha, NE, USA; 4 Department of Health, Exercise Science, and Recreation Management, School of Applied Sciences, University of Mississippi, Oxford, MS, USA

**Keywords:** College food security, College students, Environment, Food insecurity, Policy, Systems

## Abstract

College student food insecurity (FI) is a public health concern. Programming and policies to support students have expanded but utilisation is often limited. The aim of this study was to summarise the barriers to accessing college FI programming guided by the social ecological model (SEM) framework. A scoping review of peer-reviewed literature included an electronic search conducted in MEDLINE, ERIC, and PubMed databases, with a secondary search in Google Scholar. Of the 138 articles identified, 18 articles met eligibility criteria and were included. Articles primarily encompassed *organisational* (17/18) level barriers, followed by *individual* (15/18), *relationship* (15/18), *community* (9/18), and *policy* (6/18) levels. *Individual* barriers included seven themes: *Knowledge of Process, Awareness, Limited Time or Schedules, Personal Transportation, Internal Stigma, Perception of Need, and Type of Student*. Four relationship barriers were identified: *External Stigma*, *Comparing Need*, *Limited Availability Causes Negative Perceptions*, and *Staff*. Ten barrier themes comprised the organisational level: *Application Process*, *Operational Process*, *Location*, *Hours of Operation*, *Food Quality*, *Food Quantity*, *Food Desirability or Variety of Food*, *Marketing Materials*, *Awareness of the Program,* and *COVID-19 Restrictions*. Two barrier themes were identified at the *community* level, *Public Transportation* and *Awareness of SNAP*, while one barrier theme, *SNAP Eligibility and Process*, encompassed the *policy* level. Higher education stakeholders should seek to overcome these barriers to the use of food programmes as a means to address the issue of college FI. This review offers recommendations to overcome these barriers at each SEM level.

## Graphical abstract

**Figure:**
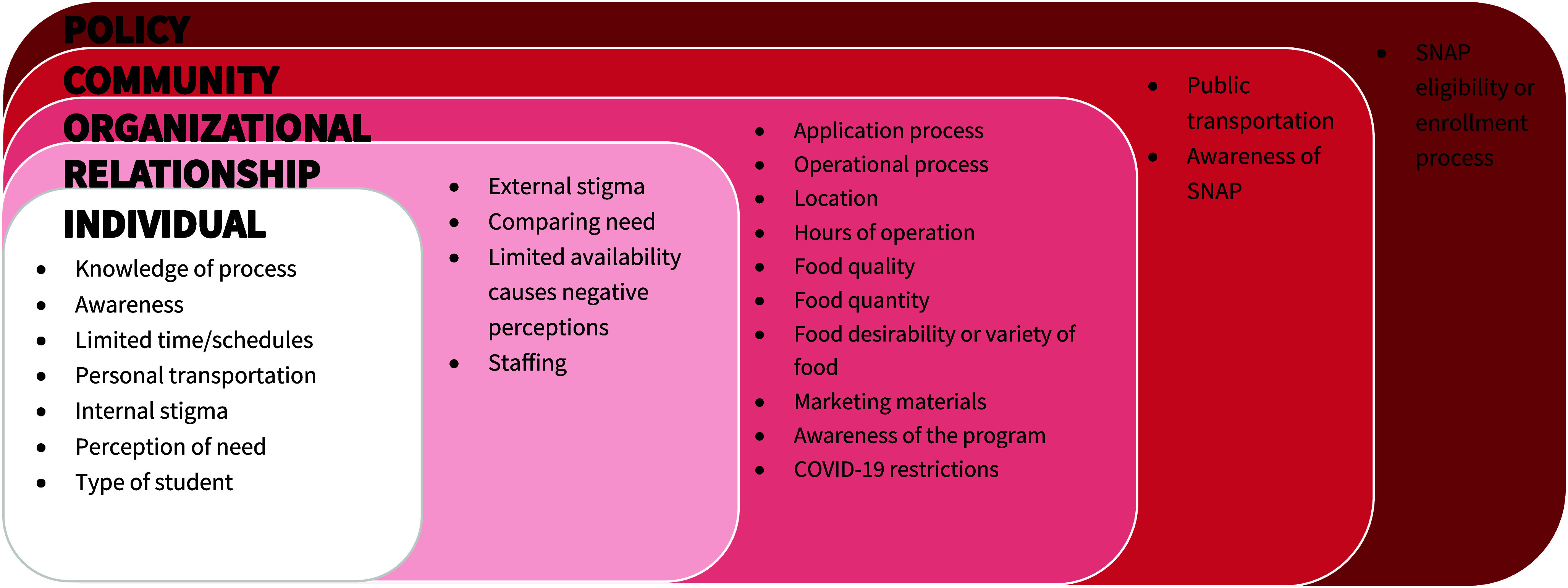

## Introduction

Research on college food insecurity (FI) has demonstrated a heightened prevalence among students and reviews have further described the academic, health, and social consequences of experiencing FI while in college.^(1,2)^ To address this, there has been an increased focus on identification of successful initiatives, programmes, and policies that universities can implement to create a culture that supports food security and health equity among students.^(3)^

Many colleges and universities report a greater availability and increased variety in the type of campus-based food resources to support food insecure students.^(4,5)^ These include food pantries, farmers markets, basic needs centres, and dedicated staff who can assist students with enrolling in federal nutrition assistance programmes.^(5)^ However, the usage of available programming is suggested to be limited among students. Barriers to utilising campus resources have been discussed^(6–8)^ and frequently include individual and operational factors such as social stigma or shame, a student’s self-identity, insufficient information on resource use policies, inconvenient availability, and inefficient marketing of available resources to students.

Beyond campus resources, students may be eligible for food and nutrition assistance programmes such as Supplemental Nutrition Assistance Program (SNAP), the largest component of the social safety net against FI in the United States.^(9)^ However, because of barriers to applying including navigating the daunting and confusing application process and confusion about eligibility requirements, many eligible students do not participate in the programme.^(10,11)^ There have been increased calls for researchers to examine which campus-based programmes are most effective in helping overcome barriers to access and utilisation of nutrition assistance programmes.^(11)^

To our knowledge, the body of literature does not include a comprehensive review that details the intra- and inter-student barriers students face when accessing resources provided at the campus, community, and/or federal levels. The purpose of this scoping review was to examine the intra- and inter-student barriers college and university students face when accessing existing programming and policies to improve their food and nutrition security. Food and nutrition security was defined in this review as equitable access to adequate quantities of quality food for optimal health. Using the social ecological model (SEM) framework, we examined programmes and policies at the campus, community, and federal government levels.

## Methods

The review was conducted in accordance with the JBI methodology for scoping reviews.^(12)^ The PRISMA Extension for Scoping Reviews (PRISMA-ScR) was used to guide the reporting of scoping review methods, process, and results^(13)^(Supplemental Materials).

### Eligibility criteria

College students at all levels (undergraduate, graduate, distance, etc) were the target population of this scoping review. College students had to be enrolled at universities or colleges in the United States. We did not exclude specific groups of students (e.g. international, students on an educational visa, etc.). The concept of interest was college student barriers when accessing food assistance programming or resources. The context for programmes included campus, community, and federal programmes intended to support food insecure college students. For the purpose of this scoping review, barriers needed to address programme utilization, thus studies that only identified barriers to food access, which in turn contributed to FI, were excluded. Articles that present the barriers to programming from the viewpoint of college students were included. Articles that included the viewpoints of only faculty, staff, administrators, or other stakeholders were excluded. Articles had to be available in English and published between January 2009 and December 2022. Our search focused on articles published after January 2009 coinciding with the publication of the first manuscript on college FI.^(2)^ Eligible articles included peer-reviewed and grey literature, including theses and dissertations.

### Search strategy

A search strategy was designed in consultation with a research librarian to identify peer-reviewed publications relevant to the research question. All search terms from a previous scoping review on college FI initiatives were used^(5)^ and expanded upon to focus on barriers. Search terms for this review included: food insecurit* or food secur*; college or university student; barrier or obstacle or issue or problem or challenge or difficult* or facilitator or motivat* or enabler and intervention* or strateg* or program* or best practice* or direct student support* or student support* or systemic reform* or practice* or protocol*. The comprehensive search occurred using three databases; MEDLINE and ERIC databases through EBSCOhost and PubMed all using similar parameters. These databases were selected based on their inclusion of nutrition and higher education-focused journals. A secondary search occurred in Google Scholar to ensure all relevant articles were included.

### Article selection

**Fig. 1.**
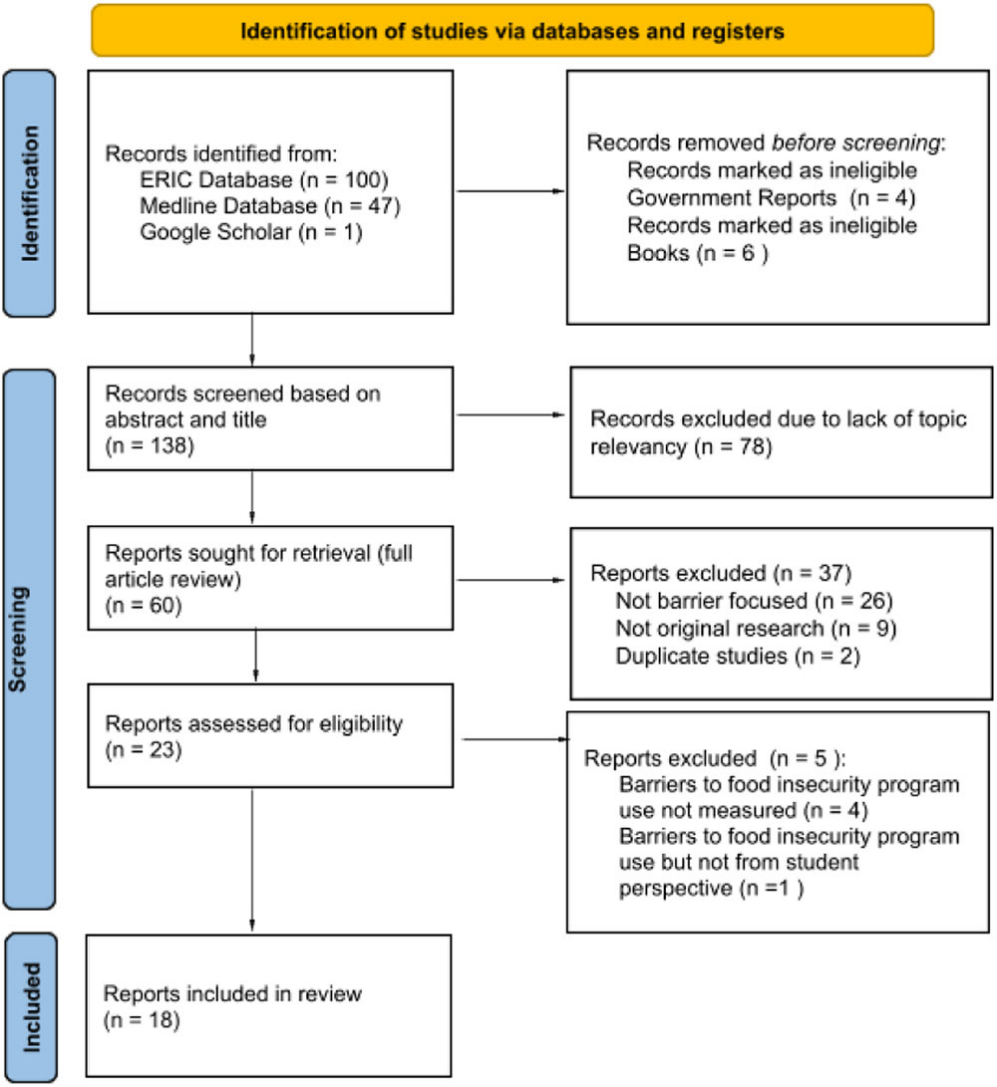
Prisma flow diagram.

All identified peer-reviewed and grey literature sources found using selected databases were uploaded to EndNote. Duplicates were removed. The authors used a three-step process to review the articles; title review, abstract review, and full-text review. To ensure inter-rater reliability, the authors met and discussed the application of inclusion and exclusion criteria. The extracted articles were divided into thirds and each author reviewed two thirds of the article titles and abstracts. Authors met to discuss any discrepancies between articles that should be kept or removed during the title and abstract review phases. Disagreements that arose were resolved through discussion, with an additional review by a third author as needed. The remaining articles were divided between the authors for full-text review followed by another meeting to discuss any articles that needed review by the full authorship team. The article selection and screening process is shown in Fig. 1 (PRISMA 2020 Flow diagram).

### Data extraction

A standard data extraction template developed by the authors in Google Sheets was used. The authors divided final articles, so each member extracted one-third of the articles. Descriptive information extracted from all articles included the full citation, study aim, sample size, research method (quantitative, qualitative, or mixed-methods), student population, institution description and whether the food programme assessed was an intervention. Additional data were extracted regarding the measures used to assess the barriers, the food resource in question (SNAP, food pantry, etc), the barriers reported, and concluding points. Any disagreements in data extract were discussed and resolved through consensus by the authorship team.

### Data synthesis and framework analysis

Extracted data related to barriers were coded by the authors. Each author independently coded the barriers for the articles in which they extracted data. The authors met to discuss the codes generated and refine the codes. Refined codes were organised by the SEM level the barrier addressed. The SEM levels used in this review align with previous health research.^(14)^ *Individual* or intrapersonal level included all barriers that may relate to one’s personal ability to utilise the food resource such as knowledge, attitudes, behaviour, or self-concept. The *relationship* or interpersonal level included barriers that related to the formal and informal social networks that prevent food resource usage such as stigma or competition with other users. *Organisational* level factors related to the food resource’s characteristics including location, hours of operation, and marketing as well as the rules and regulations for applying and using the resource. The *community* level included the context of the whole higher education institution and larger community surrounding the institution or food resource such as transportation and outreach. *Policy* level factors focused on the broader state and national policies that prevent food resource usage such as SNAP eligibility restrictions for college students. A final codebook was generated, and one author reviewed all articles using the codebook to confirm all codes had been applied to barriers identified. All authors reviewed the final codes and SEM levels identified and met to discuss until a consensus was present.

## Results

A total of 138 unique studies were screened based on abstract and title, and the full text of 60 studies were screened for inclusion in the study (Fig. 1). After review of these studies, a total of 37 studies were excluded for the following reasons: 26 did not focus on students’ barriers to accessing FI programming, 9 were not original research and 2 reported the same results as another study that was already included. The remaining 23 articles were reviewed by the study team, and an additional five articles were excluded during full article review for the following reasons (4 did not address barriers to FI programming, and one was not written from the student perspective) a total of 18 articles remained and were included in the final sample.

**Fig. 2.**
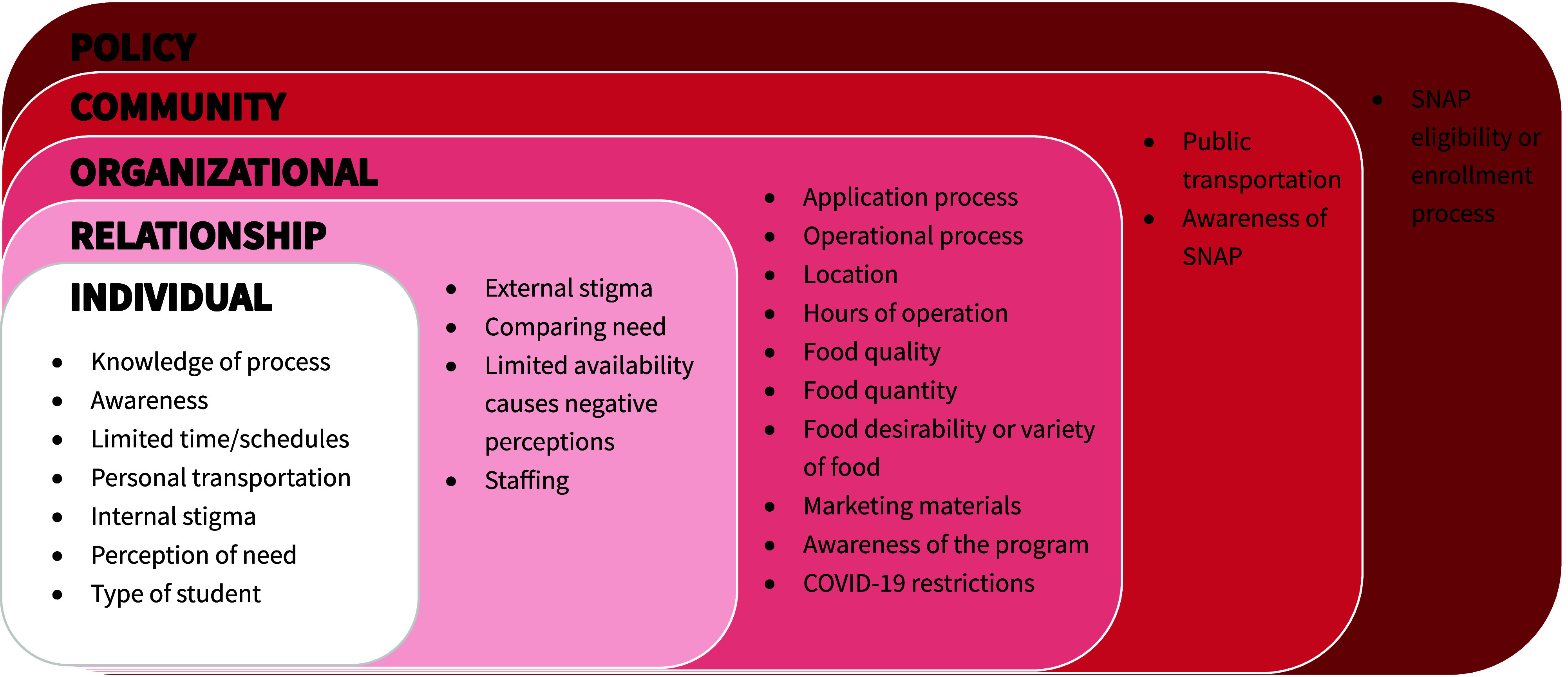
Socio-ecological model of barriers to college student food access. Abbreviations: SNAP, Supplemental Nutrition Assistance Program.

Selected study characteristics included in the final sample are presented in Table 1. All studies included in the review and applicable to our research question were published in 2020 or later, except El Zein in 2018. The recency of the literature could be attributable to the more recent focus within the field of understanding intra- and inter-student barriers students experience versus a focus on the prevalence of FI among college students which dominated the early college FI literature starting in 2009. Studies incorporated three research designs, including qualitative^(15,16,18,20,26,28–30)^ (n = 8), quantitative^(17,23,25)^ (n = 3), and mixed methods designs^(7,8,19,21,22,24,27)^ (n = 7). Two studies included an intervention.^(22,24)^ The sample size across studies ranged from 8 to 1,632. Most studies included both undergraduate and graduate students. Three studies included only undergraduate students.^(16,19,30)^ Two studies focused on a particular segment of students - social work or military connected students.^(8,18)^ Within most studies, data were collected at a single college or university. Four studies collected data from multiple colleges or universities across a region or within a university system.^(18,24,28,29)^ Figure 2 displays the identified barrier themes within studies at each level of the SEM.

**Table 1.** Study characteristics

Reference	Study design	Description of study participants (n)	Institutional description
Anderson A. *et al.* 2022^(15)^	Qualitative Semi-structured interviews	Undergraduate and graduate students (n = 30)	Large, public land grant university in the Southeast United States
Beam M. 2020^(16)^	Qualitative Semi-structured interviews	Undergraduate students (n = 8), non-traditional students	Mid-sized, public 4-year doctoral/research university.
Brito-Silva *et al.* 2022^(17)^	Quantitative Cross-sectional, non-probability, Web-based survey via email	Undergraduate and graduate students (n = 529)	Texas Women’s University
Crutchfield *et al.* 2020^(18)^	Qualitative Semi-structured interviews and focus groups	Undergraduate and graduate students (n = 16), social work students	Several campuses within the California State University System
Conrad *et al.* 2022^(19)^	Mixed Methods Cross-sectional, non-probability, Web-based survey via email. Virtual and face-to-face focus groups	Undergraduate students (n = 58, qualitative arm; n = 1159 quantitative arm)	Mississippi State University
El Zein *et al.* 2018^(7)^	Mixed Methods Cross-sectional, non-probability, Web-based survey via email	Undergraduate and graduate students (n = 899)	University of Florida
El Zein *et al.* 2022^(20)^	Qualitative Semi-structured, in-person interviews, and survey	Undergraduate and graduate students (n = 41)	University of Florida
Fortin *et al.* 2021^(21)^	Mixed Methods Focus group and interviews	Undergraduate and graduate students (n = 19, focus group; n = 11 individual interviews)	Midwestern university
Gamba *et al.* 2021^(22)a^	Mixed Methods Head-to-head crossover trial. In-person interviews	Undergraduate and graduate students (n = 30)	California State University, East Bay
Hege *et al.* 2021^(23)^	Quantitative Cross-sectional, non-probability, Web-based survey via email, social media, or tableling/flyers	Undergraduate and graduate students (n = 1632)	University of Kentucky
Hernandez *et al.* 2021^(24)a^	Mixed Methods Sequential explanatory design, randomised controlled intervention (phase 1), focus group and photo-elicitation intervention (phase 2)	Undergraduate and graduate students (n = 1000)	Two campuses within a large community college system that operates in the Houston and greater Houston, Texas area
Hiller *et al.* 2021^(25)^	Quantitative Cross-sectional, non-probability, Web-based survey via email	Undergraduate and graduate students (n = 938)	Iowa State University
Kim *et al.* 2022^(26)^	Qualitative Focus groups	Undergraduate and graduate students (n = 21)	Virginia Commonwealth University
Manboard *et al.* 2021^(27)^	Mixed Methods Four-part sequential explanatory design: (1) survey, (2) in-depth interviews, (3) photo and text elicitation; and (4) final survey^b^	Undergraduate and graduate students (n = 18)	Texas State University
Martinez *et al.* 2021^(28)^	Qualitative Semi-structured, focus groups	Undergraduate and graduate students (n = 58)	Five universities in the University of California System (Berkeley, Irvine, Merced, Santa Cruz, and San Francisco).
Richards et al 2023^(29)^	Qualitative Semi-structured, in-person interviews	Undergraduate and graduate students (n = 58)	Three universities in the western United States (Brigham Young University, Oregon State University, and the University of Hawaii-Manoa)
Schinkel et al 2023^(8)^	Mixed Methods Cross-sectional, non-probability, Web-based survey via email; semi-structured virtual focus groups via purposeful convenience sampling	Undergraduate and graduate students, military-connected students (n = 127, survey; n = 8, focus group)	University of Wyoming
Yamashiro *et al.* 2022^(30)^	Qualitative Semi-structured interviews	Undergraduate students (n = 16)	Single campus within the California State University System

a Study design involved an intervention.

b Manuscript reports on data from the first two phases (initial survey and interviews).

### Individual

Within the SEM framework, 83% of articles reviewed contained at least one *individual* level barrier that students expressed experienced when accessing FI programming. Seven barrier themes were identified at the *individual* level: *Knowledge of Process, Awareness, Limited Time or Schedules, Personal Transportation, Internal Stigma, Perception of Need,* and *Type of Student*. Of these themes, *Internal Stigma* appeared most frequently, within 11 articles of the 18 articles reviewed.^(7,8,15–20,23,27,29)^ This theme encompassed qualitative or quantitative connections to students feeling internal embarrassment or weakness that prevented them from using an available resource. The *Perception of Need* theme involved students considering it the norm to be hungry in college or downplaying their own FI by believing their situation wasn’t severe enough to warrant concern or deem them eligible to receive assistance. This theme was mentioned within eight studies.^(8,17–21,27,29)^ The *Type of Student* theme referred to any mention where a student’s perceived or actual characteristics such as living on versus off campus, being an online/distance student, their programme of study, or having international status, impacted their ability to access programmes. This theme was identified in seven of the reviewed articles.^(8,17,18,21,27,29,30)^ For example, a student that is a commuter (i.e. resides off campus) may feel that on-campus services, like a food pantry, are available only to students who live on-campus. The *Knowledge of Process* theme encompasses students being uncertain about how to enrol, access, or utilise available programming and was identified within seven of the reviewed articles.^(7,17,20,21,26,27,29)^ This theme differed from the *Awareness* theme, which was identified in five articles,^(7,17,20,26,29)^ where students were not even aware of the programme/policies available on their campus community to support them in accessing food. The theme of *Personal Transportation* encompassed student’s lack of personal transportation prevents them from accessing available programmes. This theme was mentioned within five articles.^(16,17,23,24,27)^ An example of this theme is a student who could visit an on-campus food pantry but may not have a means to get any food back to their place of residence if they do not live near the food pantry. The final individual level theme was *Limited Time or Schedules* which included five studies^(7,17,19,23,24)^ that mentioned student’s class, work, or personal schedules limited their opportunities to access available programming. This theme differs from the available hours of programming which was coded as an organisational barrier.

### Relationships

At the *relationship* level of the SEM, a student’s social circle (i.e. peers), family and campus staff influence the barriers students experienced when accessing FI programming. Four barrier themes were identified: *External Stigma, Comparing Need, Limited Availability Causes Negative Perceptions,* and *Staffing*. A majority of articles (83%) mentioned at least one *relationship* level barrier. The barrier theme of *External Stigma* appeared most frequently of the r*elationship*-level themes, appearing in 12 of the studies.^(7,8,15–18,20,21,23,27,29,30)^ This theme encompassed students feeling embarrassed when seen using available resources/programming by their peers (e.g. seen carrying bags with items from a food pantry). This theme also includes students being hesitant to share about their circumstances or asking for help from peers or staff for fear of being judged. Another common barrier at the relationship level was *Comparing Need*, which was mentioned in nine of the reviewed studies.^(7,8,15,17–21,27)^ This theme referred to students not accessing available programming due to feeling that other students needed the programmes more than they do. The *Limited Availability Causes Negative Perceptions* theme occurred in three studies.^(20,24,28)^ This theme encompassed student experiences of limited availability of foods at programming due to perceived programme abuse by peers or feelings of competition between their peers for available food. The final theme within this level of the SEM was *Staffing* which included three studies mentioning this theme.^(19,28,30)^ Studies coded with this theme included reports of students experiencing negative interactions with staff members working or managing the food resource programming which deters student’s use of the resource.

### Organisational

*Organisational* level barriers to accessing FI programming were presented in the majority of the articles (94%), and this was the level of the SEM had 10 themes. These barrier themes included *Application Process*, *Operational Process, Location, Hours of Operation, Food Quantity, Food Quality, Food Desirability or Variety*, *Marketing Materials, Awareness of the Program* and *COVID-19 Restrictions.* These themes can be conceptualised as barriers to programme uptake that are due to the ways students access the programme, components of the programme itself or how students find out about the programme.

The *Application Process* barrier occurred in five of the included studies.^(7,17,20,21,29)^ Application process describes the process to enrol in the programme, that deters the student from accessing the programme. This may include students needing to answer questions to determine their eligibility, not knowing how to enrol, or having difficulties completing the steps necessary to use the programme. One example included students saying that the application process required them to answer embarrassing questions. Additionally, college students may qualify for SNAP, but sometimes their application would be returned saying they did not qualify. The *Operational Process* barrier occurred in five of the studies^(7,17,20,22,24)^ and describes the process of using the programme that deters students from accessing the resource in the future. This may include policies or procedures that require a check in process or having to wait in line. The *Location* barrier was present in seven of the studies,^(7,8,17,24,25,27,29)^ and includes the programme being in a physical location that is inaccessible or inconvenient for students. Oftentimes this had to do with the proximity of the campus pantry to a student’s home address. The barrier of *Hours of Operation* occurred in half of the studies (nine)^(7,16,17,21,24,25,28–30)^, and this occurred when the food resource was not aligned with the student’s needs making the resource or programme inaccessible or inconvenient for students. Some pantries have limited hours of operation during weekdays, weekends or during semester breaks. Once students access the food programme or resource, another barrier to programme use was *Food Quality. Food Quality* was identified as a barrier to programme use in three studies^(17,20,30)^ and was identified when the food available is of lower quality and not deemed appropriate to consume by students accessing the programme or food resource. This may include the best-by dates or expiration dates being exceeded, fruits or vegetables with bruises, dented cans or food items that are deemed undesirable due to the visual or sensory evaluation. The barrier of *Food Quantity* was present in three studies^(20,24,28)^ and occurred when the food resources or programmes had limited foods that meet the student demand and the overall quantity of certain types of food are limited. This may include a food resource running out of food, or not having many options available. Another barrier with regard to the available food is *Food Desirability or Variety*, which was present in nine of the studies.^(15–17,20,22–24,27,28)^ This may include lack of foods to meet dietary needs, a lack of fresh options, a lack of variety in the types of food available, a lack of culturally appropriate foods, or the overall impression given by a student of not wanting to eat a certain type of food available at the food resource or programme. The barrier of *Marketing Materials* was presented in one study^(20)^ and includes when the marketing materials are unclear or use language that is perceived as negative by students which deters their use of the programme. *Awareness of the Program* was present as a barrier in six of studies^(7,15,17,19,20,26)^ and included outreach to ensure that students are aware that the food resources that exist are not sufficient. The final *organisational* barrier of *COVID-19 Restrictions* was present in one study^(27)^ and described changes to programmes during the pandemic that negatively impacted students’ ability to use the programmes.

### Community

*Community* level barriers to accessing FI programming were presented in 44.4% of articles. Two barrier themes were identified at the *community* level: *Awareness of SNAP* and *Public Transportation*. The *Awareness of SNAP* theme encompassed the outreach and efforts to engage students in federal nutrition assistance programmes being limited which prevents students from seeking support beyond campus programming. This theme, identified in six articles,^(7,15,21,26,27,29)^ was labelled at the *community* level due to the higher education institutions role in ensuring college students are aware and connected to broader programming beyond a campus context. The *Public Transportation* theme was mentioned in five articles.^(21,23,24,27,30)^ This theme included the limited availability or structure of public transportation which prevents students from being able to access programming as well as the struggle students face when having to carry food on public transportation. This barrier was noted to be exacerbated during the COVID-19 pandemic as public transportation was deemed unsafe to utilise or access to transportation was restricted.

### Policy

Roughly a third of the articles (33.3%) included student barriers at the policy level. These barriers all fell under a singular theme of *SNAP Eligibility or Process*. This theme was noted in six articles and encompassed students being ineligible due to SNAP programme rules, uncertainty about federal nutrition assistance programme eligibility, and the overall enrolment process being confusing or daunting for students.^(15,18,21,26,27,29)^ Although most studies referred to SNAP, it is important to note that one study^(27)^ also identified barriers enrolling in the Special Supplemental Nutrition Program for Women, Infants, and Children (WIC).

## Discussion

The purpose of this scoping review was to identify barriers college students face across the SEM framework that prevent the utilisation of campus, community, and federal FI resources. To our knowledge, this is the first review to systematically examine the factors that prevent college students from using the resources available to improve their food and nutrition security. The findings of this review can help guide programming improvements at the campus, community, and federal government level to improve student access to programming and address FI among college students.

### Addressing barriers at each level of the SEM

Most studies in this review addressed the *organisational* barriers students face when attempting to utilise programming on campus. Many studies described the barriers around the *Application and Organizational Process* to access food resources. University programmes may benefit from adopting best practices as provided by the Indy Hunger Network,^(31)^ which include keeping intake as simple as possible by only collecting necessary information, avoiding client embarrassment, and improving wait times.

An important component of running any programme is ensuring that the programme is evaluated to understand its effectiveness at achieving its goals. An area of future work is to evaluate campus food security programmes to understand how effective they are at addressing college student food and nutrition security.^(32)^ There are many tools available to understand how a food security programme is achieving its goals, at different phases of student (client) engagement^(33,34)^ including *food quality and variety*.^(35–38)^ This work should also include ongoing changes to improve these programmes to better address student needs and decrease student’s barriers to programme engagement. This can be difficult to do given limited resources for the evaluation process, however on college campuses it would be meaningful to engage students in this work to fulfil academic requirements.

The student perspective is crucial to include in this line of work, as students are the ones who are accessing these programmes. A community based participatory approach to programme development and evaluation can be especially successful.^(39)^ One single organisational approach will not be the solution to FI for all students, as barriers to food security differ across student groups. This review focused on articles that included information about barriers to food security programmes from the student perspective for this reason. Including student users (those who have experience with food and nutrition insecurity) in the evaluation and development of the entire programme process may remove the majority of the barriers students face. This would include obtaining student feedback about the *organisational* level barriers including the *Application Process*, *Operational Process, Location, Hours of Operation, Food Quantity, Food Quality, Food Desirability or Variety*, *Marketing Materials, Awareness of the Program*, and *COVID-19 Restrictions. Food Quantity* may always be a concern if the demand for programming exceeds its availability. However, research on barriers to programme use identifies that these programmes are underutilised by students facing food and nutrition security. Some strategies to address this *Food Quantity* barrier may include diversifying funding streams to increase funding to purchase food, partnering with student groups for food drives, and enrolling students in other food programmes they may qualify for, such as SNAP. While operationally, *Location* may be seen as challenging to address due to space being limited on college campuses, creative solutions have been achieved including satellite food pantries,^(40)^ refrigerated smart food lockers,^(41–43)^ mobile food pantries,^(44)^ and delivery services.^(45)^ Additionally, addressing some of these barriers may in turn address other barriers. For example, creating a refrigerated smart locker programme may provide students with access to higher quality and desirable food and the change in location would also address the barrier of hours of operation and awareness of the programme. As these satellite pantry locations are in different areas on campus, the programme is likely to have a greater reach based upon where students typically go for classes and meetings. Once students overcome the *individual* and *relationship* barriers that they face, barriers at the *organisational* level that are inherent in the food security programming offered on campus may limit student’s ability to access programming and improve their food security status.

At the *individual* and *relationship* level of the SEM, one of the most impactful ways to reduce barriers college and university students face when accessing existing programming and policies is to reduce stigma. Findings from several studies underscore that many students are either not aware of the severity of their own FI, may downplay it as an inherent aspect of the college experience, or may be too embarrassed to seek out help or assistance.^(19,20,29)^ Findings also suggest that students experience external stigma directed towards them from peers or staff. University culture can reduce the stigma by rejecting cultural and societal narratives that individual weakness or faults are the cause of FI.^(3)^ FI could be framed as an experience that many college students experience and that campus-based resources are available to be accessed. Campus advocacy campaigns have been suggested as an avenue to serve a dual focus of reducing stigma and raising awareness of available campus resources.^(11)^ Raising awareness of available campus resources is particularly important as many students in the studies reviewed experienced individual level barriers stemming from inadequate knowledge about enrolment and eligibility policies and procedures, accessing available programmes, or utilising offered resources. Stigma should also be considered in the context that many FI programmes and initiatives are student-led.^(4)^ These student leaders may need sensitivity training to ensure they possess the skills to deliver programs in a manner that doesn’t inadvertently contribute to stigmatisation among their peers. Higher education institutions can employ a combination of outreach, education, and dissemination efforts to reduce barriers that students experience when trying to access available resources and programming.

The demanding nature of academic commitments and part-time employment can leave college students with little time for meal planning, preparation, and consumption. This can be even more of a challenge for food insecure students. Logistical challenges at the individual level of the SEM (e.g. transportation, limited time/schedules) were also highlighted within several articles in this review. This suggests that campus-based programming should consider adapting the availability of resources to better suit the needs of students. One emerging solution for this is the use of refrigerated smart food lockers.^(43)^ Similar to the parcel package lockers used by delivery services, smart food lockers pair an online ordering system with a collection of lockers for students to retrieve items discreetly and conveniently from the campus food pantry at locations and on a schedule that work best for the student. Examples of successful food locker programmes can be found at Bunker Hill Community College^(42)^ and Frederick Community College.^(41)^

While universities and colleges often create their own community, they are also engrained in the larger community context and shared environment. To address barriers at the *community* level it is important to be inclusive of college students as part of the larger community where they most often live and work. Public transportation for college students is not a guarantee; some campuses may lack public transportation altogether^(46)^ while available public transportation at other campuses may still be inaccessible for students due to price.^(28,47)^ In fact, it has been reported that transportation costs can account for almost 11% of a college student’s budget.^(48)^ Barriers to transportation for college students have been associated with increased likelihood of experiencing FI as well as contributing to a lack of student success.^(49,50)^ Low- or no-cost public transportation for students provides an opportunity to overcome this barrier. Evidence from the City University of New York (CUNY) and Rio Hondo College support the impact of subsidised public transportation on student retention,^(50,51)^ but more research can help advocates to justify expanded programming in cities across the US. However, this fails to address barriers for students in areas with limited public transportation. Partnership between higher education leaders and local officials is vital to develop a local transportation system that supports college students while simultaneously improving access to public transportation for the whole community. Further, advocates can demand state and federal funding be delegated to expand existing infrastructure, which is suggested to reduce the transportation burden for students.^(46)^

Despite the notion that college students are part of the larger community, efforts to make them aware of nutrition assistance programmes designed to support food insecure populations locally and federally are often lacking. In particular, this scoping review identified community outreach to engage with college students about SNAP as a barrier to programme utilisation. Efforts to increase student awareness of SNAP and other federal nutrition assistance programmes have trickled upwards in recent years through the use of campus advocacy campaigns^(11,52)^ and state policy requirements,^(53)^ such as Hunger Free Campus initiatives. Yet, a majority of states have failed to require collaboration between higher education institutions and local SNAP offices to connect eligible students with the programme.^(53,54)^ As a result, many eligible students fail to enrol in the programme^(55)^ and college students remain largely unaware of nutrition assistance programmes.^(56)^ Active participation by higher education leadership and other campus stakeholders at community meetings and invitations for community leadership to serve on a campus FI task force may provide an opportunity to forge these relationships.

Even when outreach is available, students remain confused with the process which leads to avoidance in trying to enrol in federal nutrition assistance programmes. Hesitancy can result from the confusing eligibility criteria, the time commitment to navigate the system, and the lack of support to complete the process. A strong relationship between higher education stakeholders and community SNAP agents has been identified as an integral part of successful student enrolment in federal programming.^(10)^ However, lack of consistent guidance for students is present which can increase the frustration students face when attempting to enrol in these programmes.^(10)^ As a result of the 2019 US Government Accountability Office report on college FI, the Food and Nutrition Service revised its webpage on student SNAP eligibility to increase clarity,^(55)^ although it is unclear if this has helped to overcome the barriers students and state SNAP agencies face surrounding eligibility. Faculty and staff on college campuses may also be unaware of how to support student use of these programmes, adding another layer of inconsistent support for students. Thus, it is paramount that local SNAP individual agencies, along with members of the higher education community, be trained on student SNAP exemptions and the enrolment process.^(10,11)^ The disappointment that arises when taking the time to seek assistance but being denied due to technicalities, such as number of hours worked, adds an additional barrier and prevents students from seeking access to federal programming.^(57)^ Advocates have called for revised SNAP eligibility guidelines for college students to eliminate outdated exemptions which could help overcome the *policy* level barrier identified in this scoping review.^(57)^ Ultimately, federal and state policymakers’ engagement is necessary to improve the system for college students.

### Support for creating a culture of health in higher education

It has been recommended that higher education institutions work to establish a culture of food and nutrition security and that frameworks to guide this cultural shift on campus are of importance. In this review, we utilised the SEM to assess the multi-level barriers that students face. Overcoming the barriers at each level of the SEM will help to address the need to establish a culture of health on campus. However, these results can be applied to existing frameworks used in the college FI literature. Savoie-Roskos and colleagues^(3)^ utilised a justice-based Health Equity Framework^(58)^ to propose changes higher education administrators and stakeholders can make to improve health equity for food insecure college students. This framework identifies four spheres that influence health equity: Relationships and Networks, Systems of Power, Individual Factors, and Physiological Pathways. These four spheres demonstrate the interconnected factors that contribute to health inequities in society including FI on college campuses. The barriers identified in this review align with the individual factors, relationships and networks, and systems of power that must change in higher education institutions to allow all students to access programming to support their basic needs and achieve their degree. Despite student advocates championing change on campus to overcome individual and relationship barriers, addressing systems of power is often a necessary first step to ensure food security is prioritised on campus and resources are allocated to support the removal of barriers for students, including those who have been historically marginalised and excluded from campus resources.

### Strengths and limitations

This comprehensive scoping review examined over a decade of research into college FI to determine the intra- and inter-student barriers students face when accessing food security programmes and initiatives. A strength of this review was the examination of barriers at each level of the SEM. This approach has highlighted specific areas at the campus, community, and federal government levels for stakeholders to target. This review was limited to studies published in peer-reviewed journal articles and grey literature. However, information about barriers to FI programming may be available in other forms such as conference proceedings or campus resource documentation. This scoping review searched in three databases to identify articles for this review, as well as a search of an additional database following the review, however it is possible that other databases would also include eligible articles that could have been included in this review. We were specifically interested in student identified barriers to programme and resource use in this review which resulted in exclusion of studies from other stakeholder perspectives. As such, additional barriers, and potentially solutions to the barriers students face, may be identified when considering additional perspectives. Additionally, the evaluation of food and nutrition security programming in college settings is a relatively new, and emerging area of research, and as time passes, we expect a larger body of work to be available about this topic.

## Conclusion

Efforts to alleviate college FI are often student driven with support from faculty or staff. Continued awareness among students and faculty can help shift the culture on campus to create an environment that overcomes intra- and interpersonal barriers to FI resource use. However, addressing the systems of power to overcome *organisational*, *community*, and *policy* barriers will require action from higher education administration. College and university executive leadership should look to the barriers identified in this scoping review as a contributing factor to why FI continues to persist on campuses across the country. As universities implement new programmes and initiatives on their campuses, continued evaluation of the barriers that students may experience when accessing these resources is critical to ensure the effectiveness and inclusivity of their endeavours.

## Supporting information

Landry et al. supplementary material Landry et al. supplementary material

## Abbreviations

**SEM:** Social Ecological Model; **SNAP:** Supplemental Nutrition Assistance Program; **FI:** Food insecurity; **CUNY:** City University of New York.

## Supplementary material

The supplementary material for this article can be found at https://doi.org/10.1017/jns.2024.25
